# The Microbial Diversity of Traditional Spontaneously Fermented Lambic Beer

**DOI:** 10.1371/journal.pone.0095384

**Published:** 2014-04-18

**Authors:** Freek Spitaels, Anneleen D. Wieme, Maarten Janssens, Maarten Aerts, Heide-Marie Daniel, Anita Van Landschoot, Luc De Vuyst, Peter Vandamme

**Affiliations:** 1 Laboratory of Microbiology, Faculty of Sciences, Ghent University, Ghent, Belgium; 2 Laboratory of Biochemistry and Brewing, Faculty of Bioscience Engineering, Ghent University, Ghent, Belgium; 3 Research Group of Industrial Microbiology and Food Biotechnology (IMDO), Faculty of Sciences and Bioengineering Sciences, Vrije Universiteit Brussel, Brussels, Belgium; 4 Mycothèque de l'Université catholique de Louvain (MUCL), Belgian Coordinated Collection of Microorganisms (BCCM), Earth and Life Institute, Applied Microbiology, Mycology, Université catholique de Louvain, Louvain-la-Neuve, Belgium; Auburn University, United States of America

## Abstract

Lambic sour beers are the products of a spontaneous fermentation that lasts for one to three years before bottling. The present study determined the microbiota involved in the fermentation of lambic beers by sampling two fermentation batches during two years in the most traditional lambic brewery of Belgium, using culture-dependent and culture-independent methods. From 14 samples per fermentation, over 2000 bacterial and yeast isolates were obtained and identified. Although minor variations in the microbiota between casks and batches and a considerable species diversity were found, a characteristic microbial succession was identified. This succession started with a dominance of *Enterobacteriaceae* in the first month, which were replaced at 2 months by *Pediococcus damnosus* and *Saccharomyces* spp., the latter being replaced by *Dekkera bruxellensis* at 6 months fermentation duration.

## Introduction

Lambic sour beers are among the oldest types of beers still brewed and are the products of a spontaneous fermentation process that lasts for one to three years [1]. The fermentation process is not initiated through the inoculation of yeasts or bacteria as starter cultures. Rather, microbial growth starts during the overnight cooling of the cooked wort in a shallow open vessel, called the cooling tun or coolship. Lambic beers are traditionally brewed in or near the Senne river valley, an area near Brussels, Belgium. Brewing for the production of lambic traditionally takes place only during the colder months of the year (October to March), since cold nights are needed to lower the wort temperature to about 20°C in one night. The morning following the wort cooking, the cooled wort is assumed to be inoculated with a specific air microbiota of the Senne river valley and is transferred into wooden casks which are stored at cellar or ambient temperatures, *i.e.*, typically between 15 and 25°C. Subsequently, the wort ferments and the lambic beer matures in these same casks. The end product is a noncarbonated sour beer that mainly serves as a base for gueuze or fruit lambic beers. The sour character of the beer originates from the metabolic activities of various yeasts, lactic acid bacteria (LAB), and acetic acid bacteria (AAB) [2], [3].

Previous studies of the lambic beer fermentation process identified four phases: the *Enterobacteriaceae* phase, the main fermentation phase, the acidification phase, and the maturation phase, each characterized by the isolation of specific micro-organisms [2], [3]. The *Enterobacteriaceae* phase starts after 3 to 7 days of fermentation, proceeds until 30 to 40 days, and is characterized by *Enterobacter* spp., *Klebsiella pneumoniae*, *Escherichia coli* and *Hafnia alvei* as the most frequently isolated bacteria [4], along with the cycloheximide-resistant yeasts *Hanseniaspora uvarum* (asexual form *Kloeckera apiculata* [5]) and *Naumovia (Saccharomyces) dairensis* [6] as well as *Saccharomyces uvarum* (synonym *S. globosus* [7]) [2], [3]. The main fermentation starts after 3 to 4 weeks of fermentation and is characterized by the isolation of *S. cerevisiae*, *S. bayanus/pastorianus* and *S. uvarum* [2], [3]. After 3 to 4 months of fermentation, the acidification phase occurs and is characterized by the increasing isolation of *Pediococcus* spp. and occasionally *Lactobacillus* spp., while *Brettanomyces* spp. become prevalent after 4 to 8 months of fermentation [2], [3]. The final maturation phase, during which the wort is gradually attenuated, starts after 10 months of fermentation and is characterized by a decrease of LAB [2], [3]. AAB are isolated throughout the fermentation period [2], [3].

Sour beers are currently attracting interest outside Belgium, especially in the USA. In the American craft-brewing sector, American coolship ales mimic the lambic beer production method [8], and such beers are a seasonal product from craft breweries, which contrasts to traditional Belgian lambic breweries that exclusively produce lambic beers. It is thus likely that *Saccharomyces* spp., used for the brewing of other types of beers in the American craft-brewing sector, are enriched in these brewery environments [8]. A similar microbial succession as described above was recently revealed using culture-independent and culture-dependent techniques for the American coolship ales, whereby 16S rRNA gene sequence analysis was used to identify some morphologically distinct isolates [8]. Although the latter approach is widely applied as part of bacterial identification studies, it lacks resolution between many of the species belonging to the AAB, LAB, and *Enterobacteriaceae* family, and accurate species level identifications can only be obtained after subsequent sequence analysis of more variable protein-encoding genes [9]–[12]. Except for this American brewery study, previous microbial studies on lambic beers used phenotypic identification techniques only, which are nowadays known to have an inadequate taxonomical resolution for the species-level identification of yeasts, LAB, and AAB [2], [3], [13]–[17]. In addition, the discovery of novel species and of many synonymies in these groups of micro-organisms confounds the interpretation of literature data. For instance, “*Pediococcus cerevisiae*” was reported as a key organism in lambic beer fermentation, but this species name has no standing in bacterial nomenclature and has been used for at least two of the currently known *Pediococcus* species, *i.e.*, *P. damnosus* and *P. pentosaceus* [18], [19]. Such “*P. cerevisiae*” isolates likely represent *P. damnosus*, as suggested by Van Oevelen et al. [2]. Also, Kufferath and Van Laer [20] first isolated and described the yeast recognized to confer the characteristic taste to lambic beer as *Brettanomyces bruxellensis* and *B. lambicus*. After the observation of the sexually reproducing form, the name *Dekkera bruxellensis* was introduced [21]. *B. bruxellensis* and *B. lambicus* were later recognized as synonyms of the same species [22].

The present study aimed at the characterization of the microbial communities in two batches of a traditional lambic beer during the first two years of the fermentation process by means of culture-dependent and culture-independent techniques.

## Materials and Methods

### Brewery

Samples were obtained from the Cantillon brewery (http://www.cantillon.be). This brewery is the most traditional, still active, lambic brewery in Brussels and uses the same infrastructure and most of the equipment since 1900, when the brewery was founded.

### Sampling

Mash was prepared and boiled according to the brewer's recipe. After 3 h of boiling, the hot wort was pumped into the cooling tun, which was cleaned using hot water and a 500 mL sample was taken aseptically. Subsequent 500 mL samples were taken after overnight cooling in the cooling tun and 15 min; 1, 2 and 3 weeks; and 1, 2, 3, 6, 9, 12, 18 and 24 months after the transfer of the cooled wort into the multiple wooden casks; all these samples were taken from four casks of each of two batches of brews. The brews started on February 25, 2010 (batch 1), and March 23, 2010 (batch 2). Batch 1 was fermented at cellar temperature (ranging from 12°C in winter to 20°C in summer), batch 2 in a different room at ambient temperature (10–30°C). The wooden casks had a volume of approximately 400 L and had two apertures: a bung hole at the top of the cask, which was inaccessible for sampling due to the piling of the casks, and a sampling hole at the front of the cask. The latter was positioned about 10 cm above the cask bottom, plugged by a cork and was used for sampling. After removal of the cork plug, approximately 100 mL of fermenting wort were discarded before collection of the sample. Homogenization of the samples in the casks was not possible and may have introduced a sampling bias towards microbiota that settled onto the bottom of the cask and those at the wort/air interphase. Samples were transported on ice to the laboratory and were processed the same day. One cask per batch was chosen for culture-dependent sampling throughout the whole fermentation period and the microbiota of all eight casks was studied using denaturing gradient gel electrophoresis (DGGE) of the V3 region of the bacterial 16S rRNA genes and the D1/D2 region of the yeast 26S rRNA genes.

### DGGE analysis

Crude beer samples were centrifuged at 8000× *g* for 10 min (4°C) on the day of sampling and cell pellets were stored at −20°C until further processing. DNA was prepared from the pellets as described by Camu et al. [23]. The DNA concentration, purity, and integrity were determined using 1% (wt/vol) agarose gels stained with ethidium bromide and by optical density (OD) measurements at 234, 260, and 280 nm. Total DNA solutions were diluted to an OD_260_ of 1. Amplification of about 200 bp of the V3 region of the 16S rRNA genes with the F357 and R518 primers (with a GC clamp attached to the F357 primer), followed by DGGE analysis, and processing of the resulting fingerprints was performed, as described previously [24], except that DGGE gels were run for 960 min instead of 990 min. For the amplification of about 200 bp of the D1/D2 region of the 26S rRNA genes, NL1 and LS2 primers (NL1 with GC clamp) were used, as previously reported by Cocolin et al. [25]. Similarities in fingerprint patterns were analyzed by means of Dice coefficient analysis, using the BioNumerics 5.1 software package (Applied Maths, Sint-Martens-Latem, Belgium). Gels were also examined using a moving window analysis, in which the percentage change (expressed as 100% - Dice similarity) between two consecutive sample profiles was plotted as a function of time [26].

All DNA bands were assigned to band classes using the BioNumerics 5.1 software. Dense DNA bands and/or bands that were present in multiple fingerprints were excised from the polyacrylamide gels by inserting a pipette tip into the band and subsequent overnight elution of the DNA from the gel slice in 40 µL 1× TE buffer (10 mM Tris-HCl, 5 mM EDTA, pH 8) at 4°C. The position of each extracted DNA band was confirmed by repeat DGGE experiments using the excised DNA as template. The extracted DNA was subsequently re-amplified and sequenced using the same protocol and primers (but without GC-clamp). EzBioCloud and blast [27], [28] analyses were performed to determine the most similar sequences in the public sequence databases.

### Culture media, enumeration and isolation

The samples were serially diluted in 0.9% (wt/vol) saline and 50 µL of each dilution was plated in triplicate on multiple agar isolation media. The set of isolation media used was selected based on preliminary testing of samples of lambic beers of different ages by comparing DGGE profiles of the original samples with those of all cells that were harvested from the agar isolation media tested (data not shown). A total of twenty-three combinations of different growth media and incubation conditions [20°C vs. 28°C and aerobic vs. anaerobic atmosphere] were tested and this resulted in a set of 7 isolation conditions (see below), which together yielded a community profile that reflected best the diversity obtained in the DGGE profiles of the original beer samples and excluded isolation conditions that yielded redundant results.

All bacterial agar isolation media were supplemented with 5 ppm amphotericin B (Sigma-Aldrich, Bornem, Belgium) and 200 ppm cycloheximide (Sigma-Aldrich) to inhibit fungal growth and were incubated aerobically at 28°C, unless stated otherwise. Samples were incubated after plating on de Man-Rogosa-Sharpe (MRS) agar (Oxoid, Erembodegem, Belgium) [29] at 28°C aerobically and at 20°C anaerobically for the isolation of LAB. Violet red bile glucose (VRBG) agar [30], [31] was used for the isolation of *Enterobacteriaceae* and acetic acid medium (AAM) agar [32] was used for the isolation of AAB.

Yeast isolation media were first supplemented with 30 ppm ampicillin (Sigma-Aldrich), which proved inefficient to inhibit bacterial growth. All samples starting from 3 weeks in batch 1 were subcultured in the presence of 100 ppm chloramphenicol (Sigma-Aldrich). All yeast isolation media were incubated aerobically at 28°C. DYPAI (2% glucose, 0.5% yeast extract, 1% peptone and 1.5% agar; wt/vol) was used as a general yeast agar isolation medium or was supplemented with an additional 50 ppm cycloheximide (DYPAIX) to favor slow-growing *Dekkera/Brettanomyces* spp. [33]–[35]. Furthermore, universal beer agar (Oxoid) was supplemented with 25% (vol/vol) commercial gueuze (Belle-Vue - AB Inbev, Anderlecht, Belgium) as recommended by the manufacturer and was used as an additional general yeast agar isolation medium (UBAGI).

Colonies on plates comprising 25 to 250 colony forming units (CFU) were counted after 3 to 10 days of incubation and for each of the seven isolation conditions about 20–25 colonies, or all colonies if the counts were lower, were randomly picked up.

### Matrix-assisted laser desorption/ionization time-of-flight mass spectrometry (MALDI-TOF MS) dereplication and identification

Isolates were subcultured twice using the respective isolation conditions, and MALDI-TOF MS was performed using the third generation of pure cultures by means of a 4800 Plus MALDI TOF/TOF™ Analyzer (AB SCIEX, Framingham, MA, USA), as described previously [36]. In short, Data Explorer 4.0–software (AB SCIEX) was used to convert the mass spectra into .txt-files to import them into a BioNumerics 5.1 (Applied Maths) database. Spectral profiles were compared using Pearson product moment correlation coefficient and a dendrogram was built using the unweighted pair group method with arithmetic mean (UPGMA) cluster algorithm. Homogeneous clusters consisting of isolates with visually identical and/or virtually identical mass spectra were delineated. From each cluster, isolates were chosen randomly for further identification through sequence analysis of 16S rRNA genes and other molecular markers. Sequence analysis of dnaJ and rpoB genes was performed to identify members of the Enterobacteriaceae [37], [38], of the pheS gene to identify LAB [10]–[12], [39] and of dnaK, groEL and rpoB genes to identify AAB [9]. Yeast isolates were identified through sequence analysis of the D1/D2 region of the 26S rRNA gene [14] and, whenever needed, also by determination of ACT1 and/or ITS sequences [40].

All PCR assays were performed as described by Snauwaert et al. [41]. Bacterial DNA was obtained via the protocol as described by Niemann et al. [42], whereas yeast DNA was obtained using the protocol of Harju et al. [43].

### Analysis of the microbiota of the brewery environment

To analyze the microbiota of the brewery environment, samples were taken from the cooling tun, the roof above the cooling tun, the walls and ceiling of the cellar, and the outside of the casks by swabbing about 100 cm^2^ using a moist swab that was transferred into 5 mL of saline and transported to the laboratory. The inside of a cask was sampled by rinsing it with 5 L of saline. In the laboratory, 5–10 mL portions of each sample were subsequently filtered over a 0.45-µm filter that was transferred into 30 mL of MRS, VRBG, AAM, DYPAI and DYPAIX broth, each, and incubated as described above. Enrichment cultures that showed growth after 3–10 days of incubation were plated on their respective agar media and different morphotypes were selected for further analysis. Isolates were identified as described above. Additionally, the swabs and water sample were directly streaked or plated on the agar isolation media. Air samples were taken using a MAS-100 air sampler (Merck, Darmstadt, Germany) with a flow rate of 0.1 m^3^/min placed about 1 m above the floor, for one or ten minutes using yeast and bacterial agar isolation media, respectively.

## Results

### DGGE analysis

**Figure 1:**
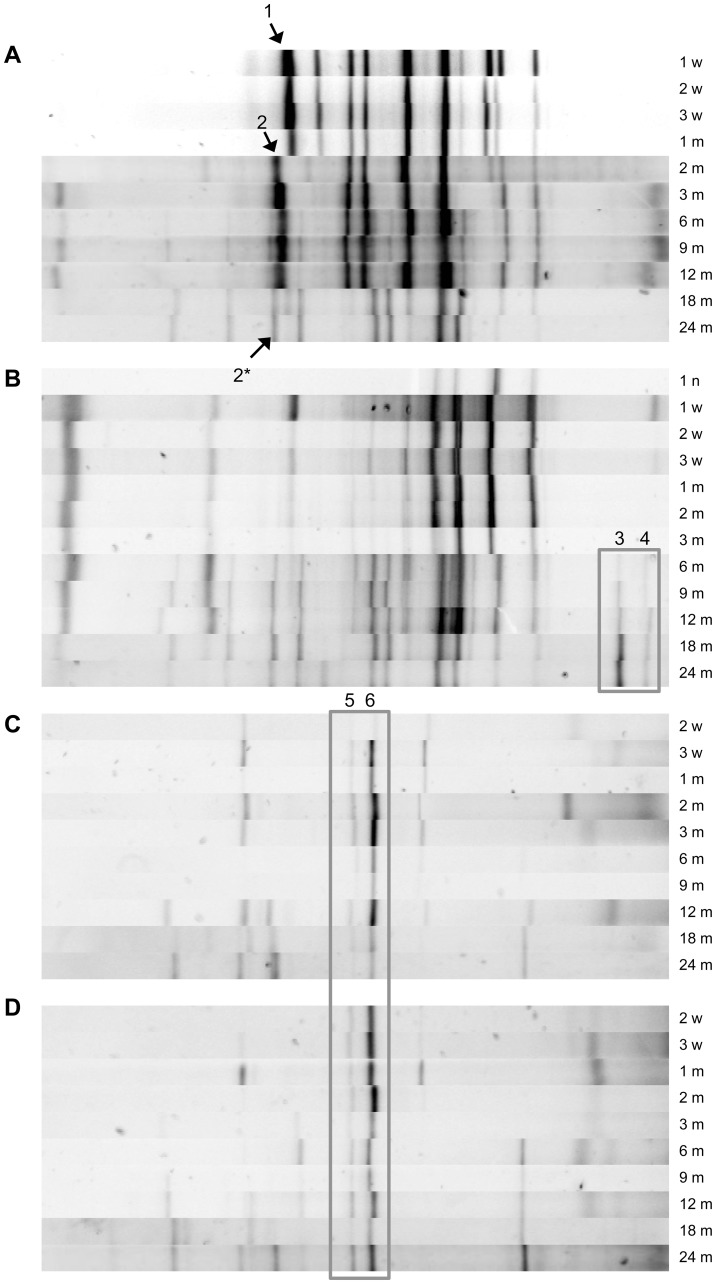
DGGE banding patterns of bacterial and yeast communities of the plated samples. DGGE banding patterns of the bacterial and yeast communities of batch 1, cask 1 (A and C, respectively) and batch 2, cask 2 (B and D, respectively) n, night; w, week(s); m, month(s). Band classes 1–6 are indicated on the figure. Samples after one night in cask 1 of batch 1 did not yield any amplicons with the V3 primer, the other casks yielded banding patterns highly similar to the pattern of the one-week sample (data not shown). Yeast community profiles were obtained from 2 weeks onwards for all casks. Nevertheless, some samples also yielded amplicons after wort transfer to the casks and after one week; these profiles were comparable to the profiles obtained after 2 weeks for all casks (data not shown).

Bacterial and yeast DNA was successfully extracted from most samples and PCR amplicons were generated subsequently. As expected, none of the cooling tun samples collected directly after boiling the wort yielded DNA (the wort temperature at the time of sampling was about 90°C). The samples of the overnight-cooled wort yielded DNA, but this was of low quality (data not shown) and no amplicons could be obtained. The first amplicons were obtained from the cask samples immediately after the transfer of the wort into the casks. For both batches, bacterial and yeast community fingerprints were generated for each of the four casks. Analysis of these community fingerprints revealed highly similar to identical community fingerprints for each sampling moment (Fig. S1). DGGE banding patterns of both bacterial and yeast communities of the casks that were used in the culture-dependent analysis of batch 1 and 2 (see below) are shown in Fig. 1.

Visual inspection of the bacterial community profiles revealed differences primarily during the first 12 months of the fermentation process, both in terms of presence and intensity of DNA bands. With the exception of two amplicons in the high % G+C region of the fingerprints (Fig. 1, band classes marked 3 and 4), the bacterial community profiles generated after 18 months were virtually identical in both batches. This bacterial community profile was reached in batch 1 after 18 months of fermentation, compared to 6 months in batch 2. The latter may be due to the incubation of batch 2 casks at ambient temperature, which was higher during the summer months compared to batch 1 casks that were incubated at more constant but lower temperatures in the cellar. In batch 1, a very dense band disappeared after 1 month of fermentation (Fig. 1, band class 1), while another band appeared in the subsequent sample taken after 2 months of fermentation time (Fig 1, band class 2).

Visual inspection of the yeast community profiles revealed more simple fingerprints comprising one to six DNA bands throughout the fermentation process. Again, the communities in both batches reached a fairly stable and highly similar composition after 6 months in batch 2 compared to 18 months in batch 1, with two amplicons in the central % G+C region of the fingerprints that were consistently present (Fig. 1, band classes 5 and 6).

**Figure 2:**
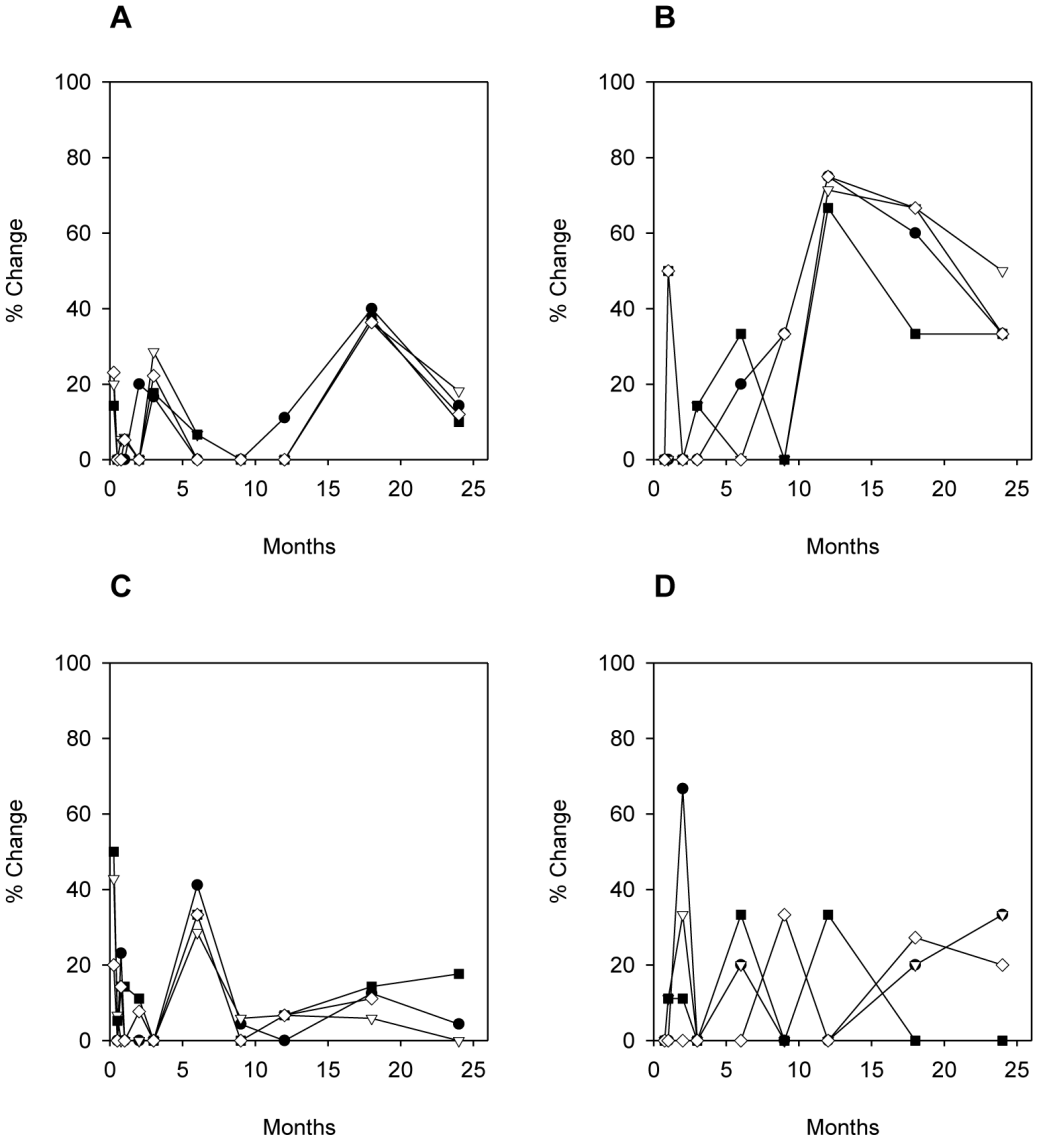
Moving windows analysis of the DGGE bacterial and yeast community profiles. Moving window analysis of the Dice-based similarity values between DGGE analyses of 4 casks from batches 1 and 2. (A) and (C) represent the bacterial diversity in batches 1 and 2, respectively, (B) and (D) visualize the yeast diversity of both batches 1 and 2. The last data point of the bacterial community profile analysis of batch 2, cask 4 was omitted due to the poor quality of the banding patterns. • Cask 1; ▽ Cask 2; ▪ Cask 3; ◊ Cask 4.

The moving window analysis of the Dice similarity values between DGGE profiles (Fig. 2A and 2C) demonstrated that the bacterial community profiles of the four casks of both batches showed a similar evolution in diversity. Consecutive samples displayed few changes. After 2 months, the appearance and disappearance of two dense bands (Fig. 1, band classes 1 and 2) resulted in a higher percentage change. The major transition in bacterial community profile appeared to occur after 18 months in batch 1, whereas the bacterial community profile changed after 6 months in batch 2 (Fig. 2A and 2C).

The moving window analysis of the yeast community profiles (Fig. 2B and 2D) revealed a higher variability. These higher percentages of change are most likely explained by the higher impact of changes in band presence or intensity in these profiles that comprised fewer bands.

A total of 64 bands (28 from yeast community fingerprints and 36 from bacterial community fingerprints) were excised (Fig. S2) and sequenced to tentatively assign these band classes to microbial taxa. Because of the short length of the sequences (about 200 bp), EzBioCloud and blast analyses resulted in genus or family level identifications only. An overview of these identification data is shown in Table S1 and demonstrates that members of the *Enterobacteriaceae* family could be detected throughout the fermentation process in both batches. Both band class 1 and 2 (Fig. 1) were assigned to members of the *Enterobacteriaceae* family. Band class 2* (Fig. 1) that migrated at nearly the same position as band class 2 was assigned to *Pediococcus*/*Lactobacillus* (which could not be distinguished by using this short rRNA gene fragment). Also, additional band classes in a higher % G+C region of the profile were assigned to LAB, which were rarely found before month 3 in batch 1 samples, but which were nearly consistently present in batch 2 samples (Table S1A and S1B). Band classes 3 and 4 (Fig. 1) were assigned to AAB, which were detected from month six onwards in batch 2 samples and primarily during year 2 in batch 1 (Table S1A and S1B). Several DNA bands of the bacterial community fingerprints were assigned to yeast taxa (Table S1A and S1B), confirming that the V3 primers were not specific for bacteria [44], [45].

The yeast band classes 5 and 6 were assigned to the genus *Saccharomyces* (Table S1C and S1D) and were present throughout the fermentation. Bands originating from other yeast taxa (*Candida*, *Dekkera/Brettanomyces, Hanseniaspora, Kregervanrija*, *Naumovia* and *Wickerhamomyces*) were found frequently, albeit on an irregular basis.

### Enumeration and identification of bacteria and yeasts

**Figure 3:**
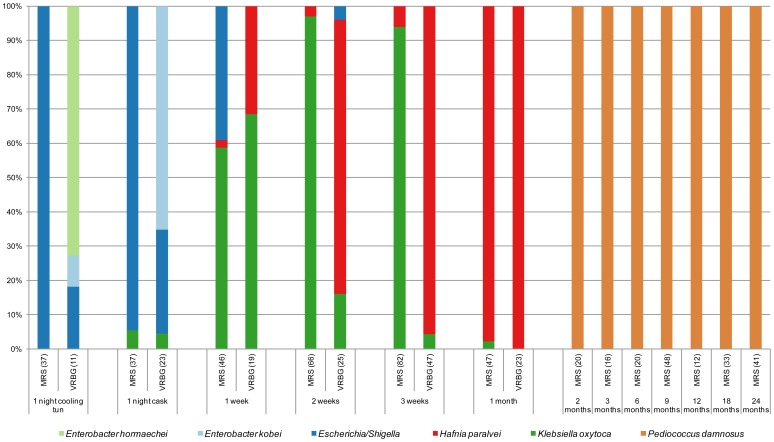
Identification of random isolates from MRS and VRBG agars of batch 1. The identification of isolates belonging to the *Enterobacteriaceae* are reported to the species level, when reliable identification by housekeeping gene sequences could be obtained. The number of isolates is given between brackets.

Table 1 presents an overview of the enumeration analyses and Table S2 presents the identifications of the MALDI-TOF MS clusters. A total of 1304 bacterial and 892 yeast isolates were obtained from the 2 batches. The freshly boiled wort did not allow microbial growth. However, both batches were spontaneously inoculated overnight in the cooling tun, as shown by the colony counts on MRS and VRBG agars, but no colonies were found on AAM agar. All cooling tun isolates (48 from batch 1 [Fig. 3] and 77 from batch 2 [data not shown]) were identified as members of the *Enterobacteriaceae* family. These bacteria were also isolated from MRS agar, which was thus not fully specific for the isolation of LAB. Both MRS and VRBG supported the growth of *Enterobacteriaceae*, but the relative species distribution differed (Fig. 3). Batch 1 isolates were identified as *Escherichia*/*Shigella* (*Escherichia coli* and *Shigella* species are extremely closely related [46] and cannot be distinguished by sequence analysis of conserved genes [47], [48]), *Enterobacter hormaechei* or *Enterobacter kobei*, whereas only the latter two were identified in batch 2 samples (31 and 46 of the 77 isolates, respectively).

**Table 1:** Results of plate counts on different agar isolation media.

Batch 1	VRBG 28°C	MRS 28°C	MRS 20°C AN	AAM 28°C	DYPAI 28°C	UBAGI 28°C	DYPAIX 28°C
Freshly boiled wort	ULD	ULD	ULD	ULD	ULD	ULD	ULD
1 night cooling tun	6.03	ULD	5.90	ULD	ND	ND	ND
1 night cask	6.51	5.25	6.42	ULD	ND	ND	ND
1 week	6.72	6.98	8.05	ULD	ND	ND	ND
2 weeks	7.73	7.68	7.77	ULD	ND	ND	ND
3 weeks	6.92	7.20	7.41	3.72	6.36	6.31	2.90
1 month	4.63	4.92	4.83	ULQ (466)	6.33	6.47	4.02
2 months	ULQ (40)	ULQ (80)	ULQ (180)	3.39	5.73	5.58	3.29
3 months	ULD	3.23	ULQ (33)	3.28	5.78	5.62	ULQ (273)
6 months	ULD	ULQ (300)	ULQ (447)	ULD	4.56	4.60	4.03
9 months	ULD	3.51	4.38	3.01	3.20	3.24	2.87
12 months	ULD	ULD	2.79	ULQ (26)	4.30	4.35	3.16
18 months	ULD	2.83	2.93	ULD	2.80	ULQ (347)	ULQ (293)
24 months	ULD	3.08	4.19	2.96	3.74	3.83	2.94
**Batch 2**							
Freshly boiled wort	ULD	ULD	ULD	ULD	ULD	ULD	ULD
1 night cooling tun	5.12	6.71	6.92	ULD	ULQ (50)	ULQ (253)	ULQ (40)
1 night cask	6.13	6.79	6.11	ULD	3.29	3.42	3.00
1 week	7.91	8.41	8.29	ULD	4.36	4.33	ULQ (140)
2 weeks	7.54	7.67	7.50	ULD	6.21	6.18	2.72
3 weeks	6.92	6.78	7.00	ULQ (40)	5.51	5.49	ULQ (120)
1 month	4.88	4.91	4.86	ULQ (270)	5.18	5.17	ULQ (67)
2 months	ULD	4.46	4.58	3.54	5.37	5.31	ULQ (13)
3 months	ULD	6.42	6.35	4.73	4.50	4.46	ULQ (353)
6 months	ULD	4.58	5.02	ULD	4.26	4.30	4.34
9 months	ULD	5.45	5.48	ULQ (40)	4.09	3.02	3.07
12 months	ULD	5.80	5.77	ULD	3.15	2.72	3.51
18 months	ULD	3.81	4.38	ULD	ULQ (173)	ULQ (300)	ULQ (240)
24 months	ULD	4.07	4.26	ULQ (66)	3.08	3.18	3.18

VRBG agar was used for the growth of *Enterobacteriaceae*, MRS agar for the growth of LAB, AAM agar for the growth of AAB, DYPAI and UBAGI agars were used as global yeast growth media and DYPAIX agar was used to favor the growth of *Dekkera* species. Values represent log CFU/mL. ULD: under limit of detection; ULQ: under limit of quantification (the estimated CFU/mL is provided between brackets); ND: no data.

*Enterobacteriaceae* counts reached up to 10^7^–10^8^ CFU/mL after one to two weeks of fermentation. A total of 415 isolates from batch 1 samples taken during the first month were identified. *E. hormaechei* was no longer isolated after the transfer of the wort into the cask (performed 15 min after the sampling of the cooling tun), whereas *Klebsiella oxytoca* was then first isolated (Fig. 3). In the following weeks, the number of isolates identified as *Escherichia*/*Shigella* and *E. kobei* decreased, while the numbers of *Hafnia paralvei* and *Klebsiella oxytoca* isolates increased until the end of the first month, after which *Enterobacteriaceae* were no longer isolated. In batch 2, from which a total of 398 isolates were identified, a similar evolution was found: the major occurrence of *H. paralvei* from week 1 onwards was confirmed and members of the *Enterobacteriaceae* were again no longer isolated after one month of fermentation (data not shown). However, batch 2 *Enterobacteriaceae* were more diverse and included also *Citrobacter gillenii* and *Raoultella terrigena* (data not shown).

From months 2 until 24, *Pediococcus damnosus* was consistently the only micro-organism isolated from MRS agar (batch 1 [Fig. 3]; batch 2, n = 124 [data not shown]). The bacterial counts on MRS agar remained stable at about 10^4^ CFU/mL until the end of the fermentation. Colony counts on AAM agar were generally low (below 10^4^ CFU/mL; Table 1). AAM counts of the samples up to 3 months of fermentation were influenced by the presence of yeasts, which was due to the apparent loss of activity of amphotericin B under acidic conditions [49]. Amphotericin B was also reported to be unstable in other media with a composition similar to AAM [50]. A combination of amphotericin B and cycloheximide was subsequently found to be more effective in inhibiting yeast growth under all isolation conditions used.

AAB were isolated from batch 1 samples at 9 and 24 months (n = 35) and from batch 2 samples at 3, 9 and 24 months (n = 17). All but one of the isolates were identified as a novel *Acetobacter* species, for which the name *Acetobacter lambici* has been proposed [51]. One batch 2 isolate represented a novel *Gluconobacter* species, for which the name *Gluconobacter cerevisiae* has been proposed [52]. This erratic isolation of AAB was not in accordance with the consistent presence of AAB-derived DNA bands in the DGGE profiles from 6 months of fermentation onwards in batch 2 (Fig. 1).

**Figure 4:**
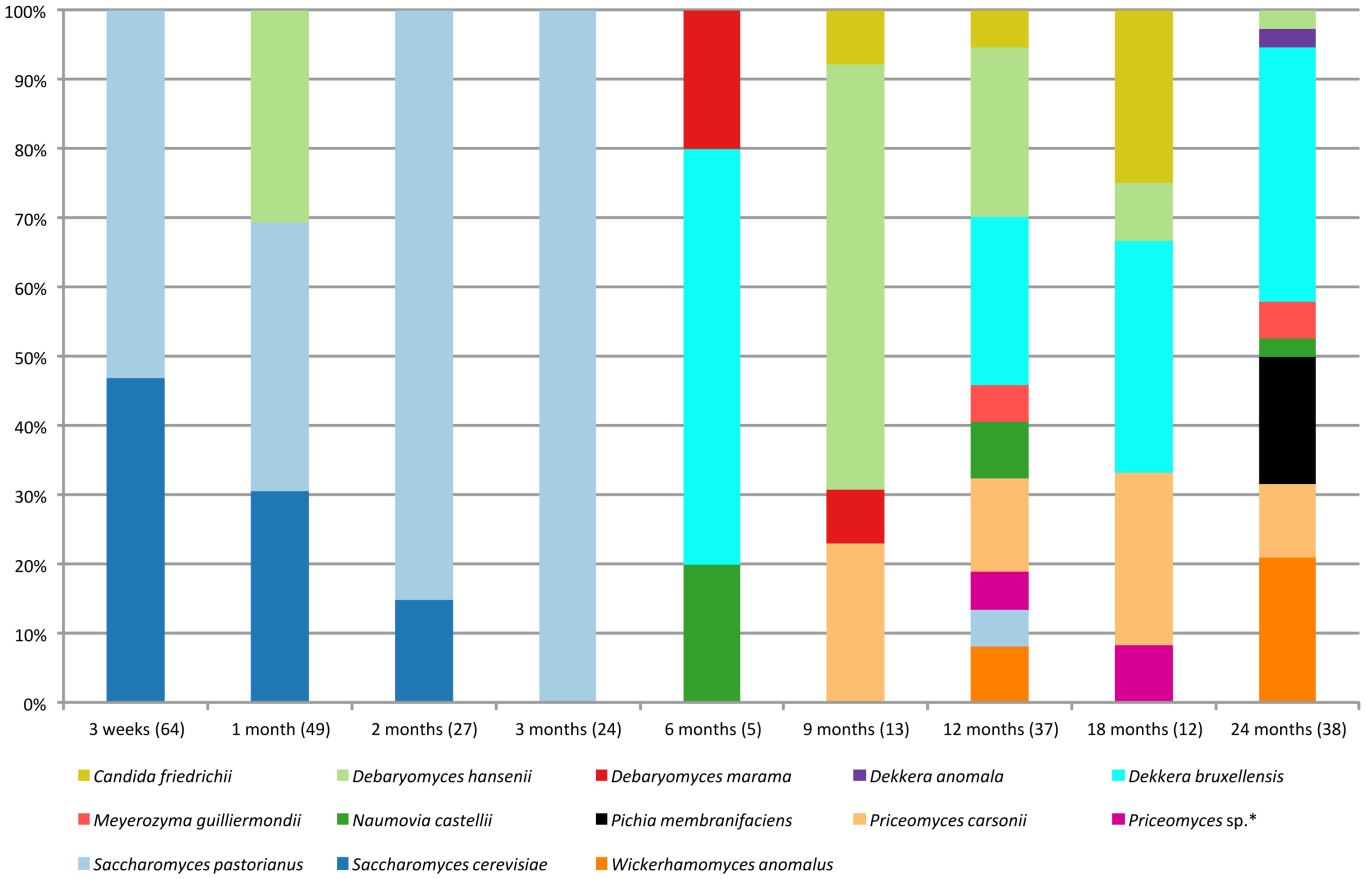
Identification of random isolates from DYPAI and UBAGI agars of batch 1. The number of isolates is given between brackets. *One yeast cluster from MALDI-TOF MS profiles could not be identified unambiguously (Table S2).

An overview of the identified yeast species of batch 1 is graphically represented in Fig. 4 and Fig. S3. Isolation and accurate enumeration of yeasts during the first two weeks of fermentation of batch 1 was not possible, due to an insufficient suppression of bacterial growth. In batch 2 samples (data not shown) yeasts could not be detected in the wort after one night in the cooling tun, but increased in numbers directly after the wort was transferred into the casks not more than 15 min after the cooling tun was sampled. Maximal counts (10^6^ CFU/mL) were reached after 2 weeks to 1 month of fermentation. *Debaryomyces hansenii* (17/18 isolates examined) and *S. cerevisiae* (1/18) were the sole species isolated directly after the transfer of the wort into the cask in batch 2. *S. cerevisiae* (22/44), *S. pastorianus* (21/44) and *Naumovia castellii* (1/44) were isolated after 1 week of fermentation. The relative number of *S. pastorianus* isolates increased further during the first three months of fermentation (a total of 198 isolates examined), until it was the only yeast species isolated on DYPAI and UBAGI agars after 2 months (32 isolates examined). After 3 months, *S. pastorianus* was still the predominant yeast (30/31); one isolate was identified as *N. castellii*.

The same trend occurred during the first three months of fermentation of batch 1 (Fig. 4). *S. cerevisiae* and *S. pastorianus* were the most prevalent species and the latter one was the only yeast species present after three months. Yeast counts on DYPAIX agar were initially lower compared to DYPAI and UBAGI agars, but were comparable from 6 months onwards. The few DYPAIX isolates that were obtained from samples after 2 months (batch 2) or 3 months (batches 1 and 2) failed to grow on the same growth agar medium upon subculture, indicating that there were no cycloheximide-resistant yeast species present in these samples (Fig. S3). DYPAIX isolates obtained from samples of the first 2 months of batch 1 included *N. castellii*, *Kazachstania servazzii* and *Db. hansenii* (Fig. S3), whereby the former was the only species isolated in the first month of batch 2 (n = 58, data not shown).

*Saccharomyces* spp. were not isolated in large numbers after 6 months of fermentation, while *D. bruxellensis* was isolated at this point for the first time. *D. bruxellensis* was the major yeast species isolated from DYPAI and UBAGI agar media from 6 months until the end of the fermentation of batch 2 (n = 102, data not shown) and the only yeast species isolated from DYPAIX agar in the same period (n = 82). The cultivated yeast diversity in batch 2 was low compared to batch 1 (see below) and the three yeast media yielded the same species diversity from 6 months onwards.

The yeast species distribution in batch 1 samples after 6 months of fermentation (Fig. 4) was more complex than that of samples of the same age in batch 2. The most frequently cultivated species were *D. bruxellensis, Db. hansenii*, *Priceomyces carsonii* and *Wickerhamomyces anomalus* along with other species in lower numbers (Fig. 4 and Fig. S3). In contrast to batch 2 where the three yeast agar isolation media yielded the same species diversity from 6 months onwards, the species diversity recovered from different yeast agar isolation media in batch 1 was not comparable. For example, *D. bruxellensis* was not detected on the non-selective yeast agar media in batch 1 after 9 months, but was detected at this sampling point on DYPAIX agar (Fig. 4 and Fig. S3). The use of DYPAIX agar allowed isolating some unusual species from batch 1, such as *Candida patagonica* and *Yarrowia lipolytica* (Fig. S3), of which the latter has never been associated with a beer fermentation process. The total yeast and bacterial counts were similar in both batches after 24 months at about 10^3^–10^4^ CFU/mL (Table 1).

### Air and brewery environment

None of the directly plated samples yielded growth. A total of 139 isolates from the brewery environment were picked up from the bacterial and yeast agar isolation media after enrichment and were identified through MALDI-TOF MS and sequence analysis of 16S rRNA genes or other molecular markers as described above (Table 2). Several species or taxa that were previously isolated during the fermentation process as described above were also found in environmental samples. *E. hormaechei* and *Escherichia*/*Shigella* were isolated from the cellar air. *Raoultella terrigena*, *Pichia membranifaciens*, *Debaryomyces marama* and *Db. hansenii* were isolated from the inside of a cask. The latter species was also isolated from the ceiling, the attic and cellar air, along with *S. pastorianus*, *Meyerozyma guilliermondii*, *Candida friedrichii* and *Wickerhamomyces anomalus*. The latter species was also found on the outside of a cask. A considerable number of additional micro-organisms that were not detected during the fermentation process were also isolated from environmental samples. These included species previously related to beverage fermentation or spoilage, such as *Brettanomyces custersianus* [53], *Pediococcus pentosaceus* [54], *Lactobacillus malefermentans* [55] and *Acetobacter cerevisiae* [56].

**Table 2:** Overview of micro-organisms isolated from the brewery environment and their isolation sources.

Identification	Accession number	Accession number highest hit	Similarity	Present in fermentation	Air attic before cooling	Cooling tun	Roof	Air attic after cooling	Air cellar	Cellar ceiling	Cellar wall	Cask outside	Cask inside
**Bacteria** a													
*Acetobacter cerevisiae* b		KF537492	100%										+
*Aerococcus urinaeequi*		D87677	100%		+								
*Bacillus licheniformis*		AE017333	100%				+						
*Enterobacter hormaechei*				+					+				
*Enterococcus faecium* c	KJ186124	AJ843428	97%		+	+							
*Escherichia/Shigella*				+					+				
*Hafnia alvei*		M59155	100%										+
*Lactobacillus curvatus*		AJ621550	100%						+				
*Lactobacillus malefermentans*		BACN01000105	100%									+	
*Lactobacillus nenjiangensis* c	KJ186125	HF679044	99%		+								
*Leuconostoc mesenteroides*		CP000414	100%		+				+		+	+	
*Leuconostoc pseudomesenteroides*		AEOQ01000906	100%		+								
*Pediococcus pentosaceus* c		AM899822	100%		+								
*Pseudomonas azotoformans*		D84009	100%		+								
*Pseudomonas libanensis*		AF057645	100%		+								
*Pseudomonas psychrotolerans*		AJ575816	100%		+								
*Rahnella aquatilis*		CP003244	100%										+
*Raoultella terrigena*				+									+
*Staphylococcus hominis*		X6601	100%		+			+	+				
**Yeasts** d													
*Brettanomyces custersianus*		DQ406717	100%										+
*Candida friedrichii*				+	+								
*Candida pomicola*		AF245400	100%										+
*Cryptococcus heveanensis*		AF075467	100%										+
*Cryptococcus magnus*		AF181851	100%		+			+					+
*Debaryomyces hansenii*				+	+				+	+			+
*Debaryomyces marama*				+									+
*Meyerozyma guilliermondii*				+	+								
*Pichia membranifaciens*				+									+
*Priceomyces* sp.*				+					+				
*Saccharomyces pastorianus*				+					+				
*Trichosporon gracile*		JN939453	100%		+				+				
*Trichosporon cutaneum*		AF075483	100%									+	
*Wickerhamomyces anomalus*				+	+				+			+	

The bacteria and yeasts present in the fermentation were identified based on their MALDI-TOF MS spectra. *One yeast cluster from MALDI-TOF MS profiles could not be identified unambiguously (Table S2).

a Identification is based on 16S rRNA gene sequence.

b Identification is based in *rpoB* sequence.

c Identification is based in *pheS* sequence.

d Identification is based on D1/D2 26S rRNA gene sequence.

## Discussion

Serious limitations of the few available microbiological studies of the lambic beer fermentation process are the rather low numbers of isolates identified using biochemical methods only [2], [3]. Recent polyphasic taxonomic studies revealed that phenotypic identification approaches alone have an inadequate taxonomical resolution for the accurate species level identification of these micro-organisms [10], [14]–[17], [38]. Therefore, the present study revisited the microbiology of the lambic beer fermentation process of the most traditional lambic brewery (Cantillon) in Belgium and identified and monitored the microbiota using MALDI-TOF MS as a high-throughput dereplication technique. This allowed to compare numerous fingerprints and to reduce these isolates to a non-redundant set of different species that were further identified using an array of DNA sequence-based methods [15], [57]. This approach allowed a more in depth analysis of the culturable microbiota of this ecosystem and resulted in the isolation and description of two novel AAB species, *i.e.*, *Acetobacter lambici* and *Gluconobacter cerevisiae* [51], [52]. The former species was even the most frequently isolated AAB species during the lambic fermentation process of Cantillon. The present study also used DGGE profiles of variable prokaryotic and eukaryotic rRNA gene regions to identify and monitor the microbial communities in two batches of lambic beer during a two-year fermentation period at Cantillon.

In both lambic batches, members of the *Enterobacteriaceae* were isolated during the first month, which corresponded to previous studies on Belgian lambic and American coolship ales [2], [3], [8]. The bacteria identified included *E. hormaechei*, *E. kobei*, *Es. coli*, *H. paralvei*, *K. oxytoca*, *Citrobacter gillenii* and *R. terrigena*, from which some of these were already detected in the cooling tun sample, suggesting their origin from the cooling tun environment. Remarkably, DNA from members of the *Enterobacteriaceae* family was detected in the DGGE experiments throughout the two-year fermentation period. This suggests that DNA from these cells persisted for a long time or, alternatively, that these bacteria remained present in a viable but non-culturable form, even under conditions to which *Enterobacteriaceae* are susceptible, *i.e.*, pH<4.0 and ethanol concentrations over 2.0% [58]. This has also been seen during cocoa bean fermentation [59], [60]. Yeast isolations during the first three months yielded *Saccharomyces* spp., but no *Hanseniaspora* spp., as expected from previous studies [2], [3]. However, *Hanseniaspora* spp. were detected by DGGE profiles over several months in both batches. Species of this genus are frequently found in spontaneously fermenting fruit and their preparations, and a positive contribution to wine flavor development is increasingly recognized (*e.g.*, Medina et al. [61]).

After the initial *Enterobacteriaceae* phase, the effects of ethanol production by the main fermentation were reflected in the dominance of *P. damnosus* at two months, along with some AAB (primarily *Acetobacter lambici*) that were occasionally isolated. AAB may survive in the cask due to the diffusion of oxygen through the wood [62], [63] or the short vacuum-releasing opening of the bung hole during sampling. Similarly, AAB seem to survive the anaerobic phase of cocoa bean fermentations [59], [60]. The irregular isolation of AAB may suggest that they are also present in a viable but non-culturable form that could be reversed when oxygen becomes available, as for example in wine production [64]. In both batches, *P. damnosus* remained present throughout the fermentation process and these bacteria were accompanied by *D. bruxellensis* after the decrease of *Saccharomyces* spp. Remarkably, no other LAB were isolated, while *Lactobacillus* spp. and other LAB species have also been isolated from American coolship ales recently [8].

The culture-independent detection of micro-organisms by DGGE was useful to observe the similar succession of microorganisms in each of the four casks of both lambic batches, and to visualize the relative stability of community profiles over time and their homogenization in the two batches at the advanced stage of the fermentation, but it confirmed some of the established pitfalls of this methodology. For instance, some cultivated yeast genera were not detected by DGGE (*Debaryomyces, Kazachstania, Meyerozyma, Pichia, Priceomyces, Yarrowia*), while other genera were detected by DGGE but not cultivated (*Hanseniaspora, Kregervanrija*). Also, some organisms were detected by DGGE before appearing in culture or after having disappeared from cultures, such as *Enterobacteriaceae* which were detected throughout the sampling period. Similar observations using T-RFLP and barcoded amplicon sequencing were made in spontaneous fermentations of American coolship ales [8]. Cultivation experiments too can be strongly biased, for instance, by the presence of VBNC cells, the selection of the culture media in the experiment design and by culture media that favor specific organisms. Therefore, a combination of multiple complementary techniques including both culture-based and culture-independent methods and a cautious interpretation of the results remains the best approach for microbial diversity analyses [65].

The microbial community analyses of the present study did not provide evidence for an extended acidification phase [3], as after six months *P. damnosus* and *D. bruxellensis* were both present and *Saccharomyces* spp. were no longer isolated. In addition, neither lambic batch showed a clear decrease of LAB. Pending a detailed analysis of the microbial metabolites and other biochemical characteristics, the data of the present study suggest that the acidification took place rapidly at the transition from the main fermentation phase to the long maturation phase, as was also found for American coolship ale fermentations [8].

The two nearly simultaneously fermented wort batches were inoculated by micro-organisms present in the brewery air, equipment or casks. As discussed above, members of the *Enterobacteriaceae* family were present in the wort before its transfer into the casks. These rather adventitious bacteria, *S. pastorianus* and some other yeast species, may have at least partially originated from the brewery air, but the present study failed to isolate the key micro-organisms *P. damnosus, S. cerevisiae* and *D. bruxellensis* from environmental samples. These micro-organisms were either missed by the sampling protocol or were concealed in niches that were not sampled. Examples of such niches are biofilms in and the pores of the wooden casks. Micro-organisms may have penetrated and effectively be immobilized and protected from washing steps in the wood of the cask, as demonstrated by Swaffield et al. [66], [67]. All casks had been used for lambic production before, preceded by their use in different fermentations, mostly red wine, so they could have retained specific microbiota in spite of cleaning procedures after previous fermentations [66], [67].

This study generally confirmed and extended the microbial diversity and succession known from previous accounts of lambic beers. The more than 2000 microbial isolates from two fermentation batches of the present study showed diverse members of the *Enterobacteriaceae* family during the first month, and *S. cerevisiae* and *S. pastorianus* from the first week until two and three months, respectively. No LAB were recovered during this first phase, which was previously denoted as the ‘mixed acid fermentation’. The main fermentation was characterized by *Saccharomyces* spp. and the completion of the shift from *Enterobacteriaceae* to *P. damnosus*, the latter being isolated from 2 months onwards. The increase of LAB in months 2 and 3 and the concomitant decrease of *Saccharomyces* spp. was followed by the highly acid- and ethanol-resistant *D. bruxellensis,* which dominated from 6 months onwards together with *P. damnosus*. *Hanseniaspora* spp. that were previously reported in the first fermentation weeks were not isolated, but their presence was evidenced by DGGE analyses. The role of these and other taxa, such as *N. castellii* and *Kazachstania* spp., both also seen in lambic beer fermentations before, is not known.

Despite apparent differences in the microbial diversity, both batches examined reached similar community profiles at the end of the fermentation. The time needed to reach these final community fingerprints differed between the two batches and it is likely that the lower ambient temperature in the localization of batch 1 explains both the longer period needed to reach the characteristic community fingerprints as well as the larger diversity observed in later phases of the fermentation process.

## Supporting Information

Figure S1 Overview of intra-batch DGGE banding pattern differences. Overview of the differences in banding profiles for the DGGE analysis of 4 different casks (C1, C2, C3 and C4) within the same fermentation of batches 1 (B1) and 2 (B2). (A) DGGE banding patterns of the bacterial communities after 3 weeks (3 w), 6 months and 18 months of fermentation; (B) DGGE banding patterns of the yeast after 2 months (2 m) 6 months (6 m), 12 months (12 m), 18 months (18 m) and 24 months (24 m) of fermentation.
(TIF)

Figure S2 Overview of the excised DGGE bands for identification. DGGE banding patterns of the bacterial and yeast communities of batch 1, cask 1 (A and C, respectively) and batch 2, cask 2 (B and D, respectively) n, night; w, week(s); m, month(s). Band classes 1–6 are indicated on the figure. The excised bands are indicated in red and identifications based on the derived DNA sequences of these bands can be found in Table S1E.
(TIF)

Figure S3 Identification of random isolates from DYPAIX agar of batch 1. Empty bars represent isolates that could not be recovered after isolation. The number of isolates is given between brackets.
(TIF)

Table S1 Occurrence of microbial taxa as identified through sequence analysis of V3 and LSU DGGE bands. (A) and (B) summarize the identifications from the V3 DGGE analyses from batches 1 and batch 2, respectively. (C) and (D) represent the LSU DGGE identifications for batches 1 and 2, respectively. +: taxon is present. (E) represents the identifications of the excised DNA bands.
(XLSX)

Table S2 Overview of MALDI-TOF MS clusters and the identifications of the representative isolates. The number of isolates in each MALDI-TOF MS cluster is given in parentheses. The accession number of the cluster representative sequence is given when sequence similarity with a known sequence was below 100%. B: bacterial MALDI-TOF MS cluster, Y: yeast MALDI-TOF MS cluster.
(PDF)
