# Supplementary figures and images for: The Microbial Diversity of Traditional Spontaneously Fermented Lambic Beer
Source: PLoS One. 2014 Apr 18;9(4):e95384. doi: 10.1371/journal.pone.0095384 (PMC3991685; doi:10.1371/journal.pone.0095384)

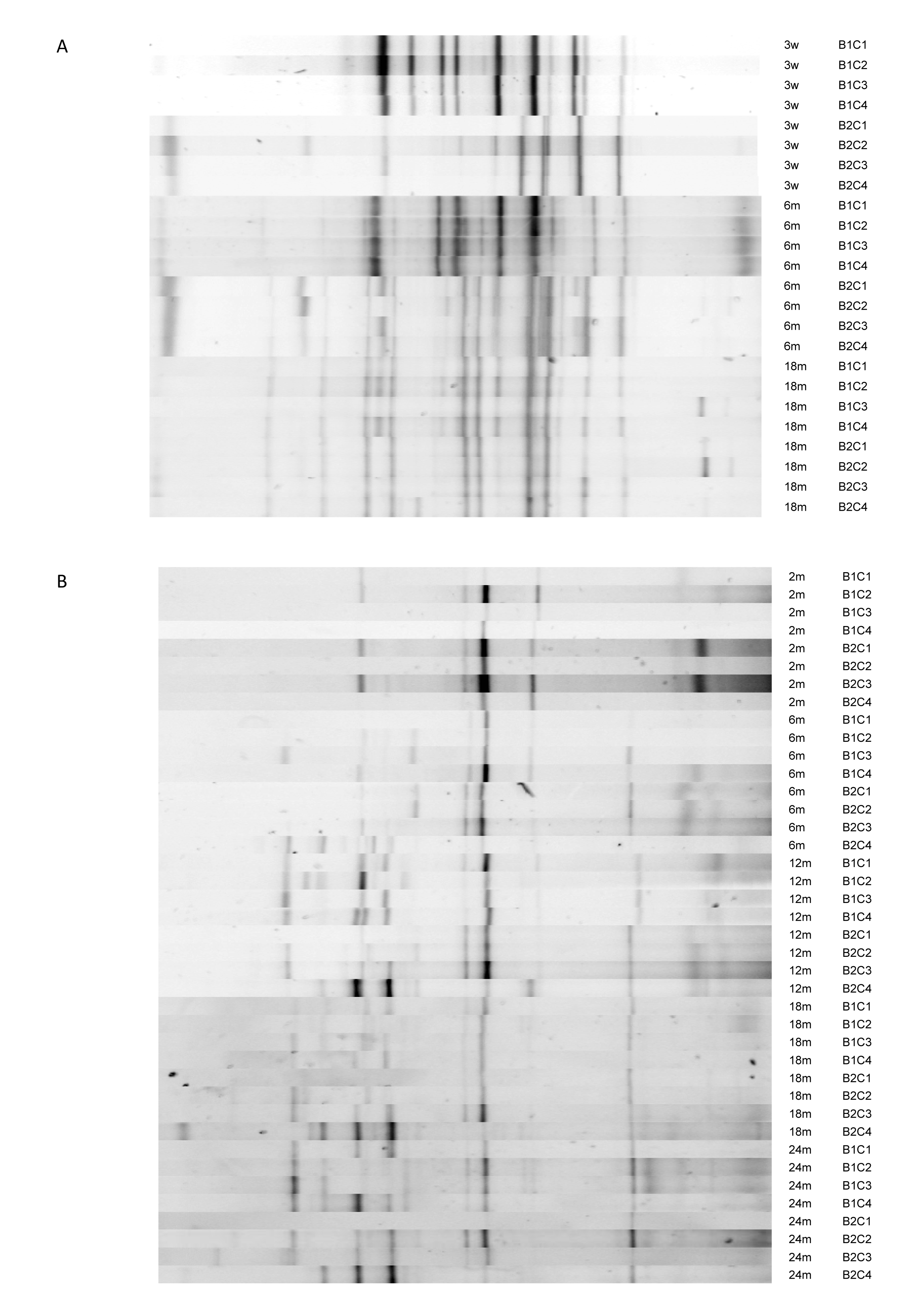

Supplement: Figure S1 — Overview of intra-batch DGGE banding pattern differences. Overview of the differences in banding profiles for the DGGE analysis of 4 different casks (C1, C2, C3 and C4) within the same fermentation of batches 1 (B1) and 2 (B2). (A) DGGE banding patterns of the bacterial communities after 3 weeks (3 w), 6 months and 18 months of fermentation; (B) DGGE banding patterns of the yeast after 2 months (2 m) 6 months (6 m), 12 months (12 m), 18 months (18 m) and 24 months (24 m) of fermentation. (TIF) [file pone.0095384.s001.tif]

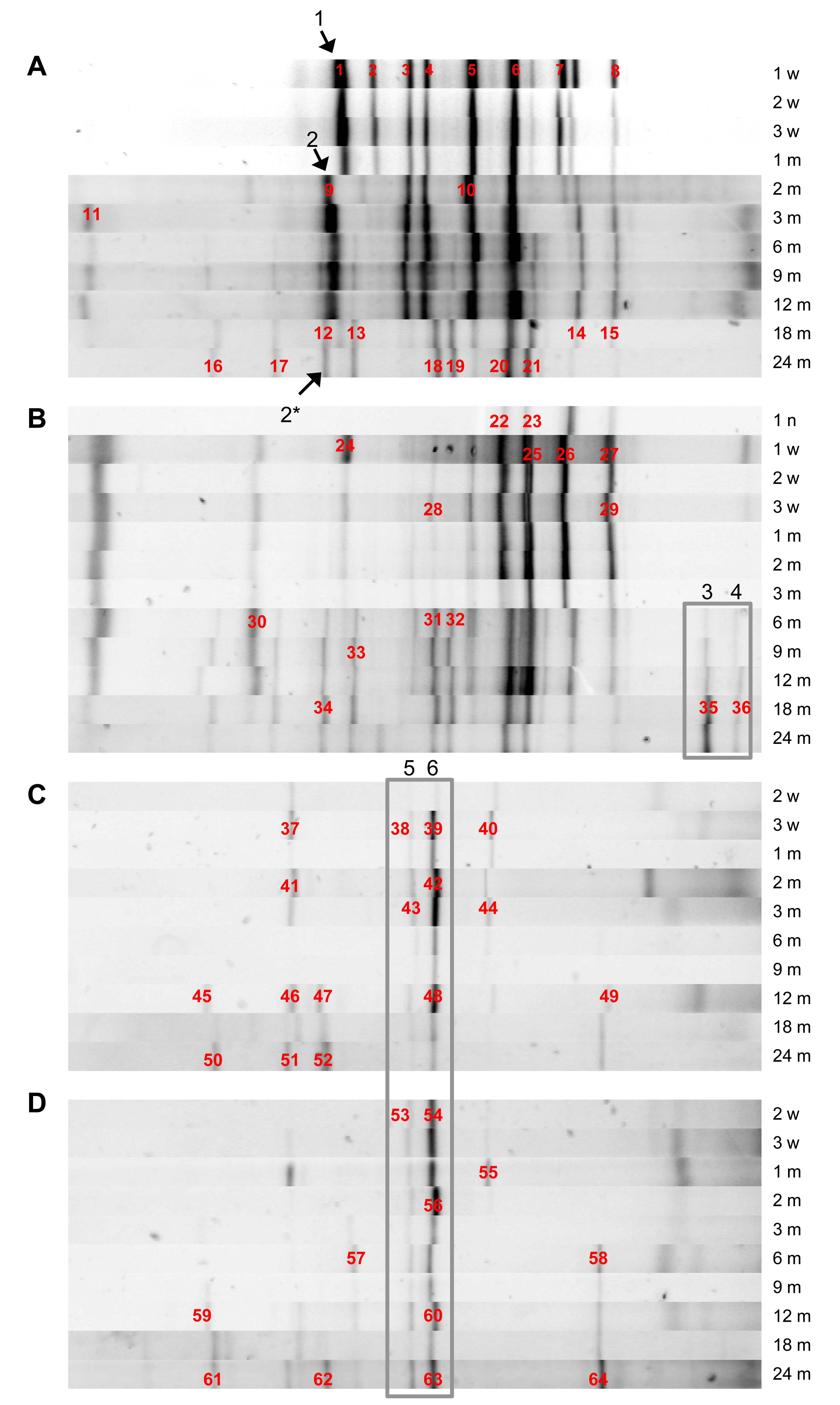

Supplement: Figure S2 — Overview of the excised DGGE bands for identification. DGGE banding patterns of the bacterial and yeast communities of batch 1, cask 1 (A and C, respectively) and batch 2, cask 2 (B and D, respectively) n, night; w, week(s); m, month(s). Band classes 1–6 are indicated on the figure. The excised bands are indicated in red and identifications based on the derived DNA sequences of these bands can be found in Table S1E. (TIF) [file pone.0095384.s002.tif]

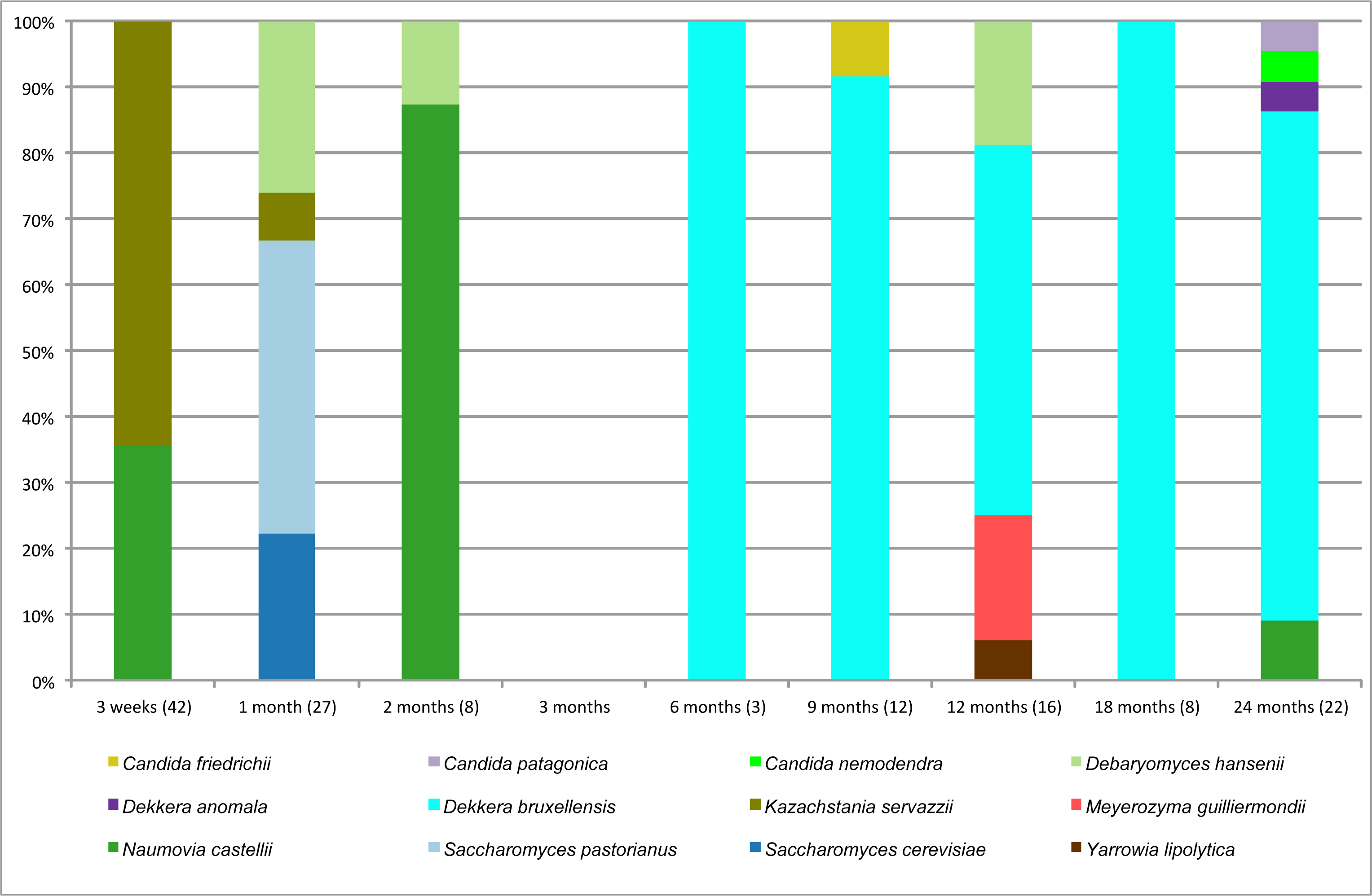

Supplement: Figure S3 — Identification of random isolates from DYPAIX agar of batch 1. Empty bars represent isolates that could not be recovered after isolation. The number of isolates is given between brackets. (TIF) [file pone.0095384.s003.tif]
