# Supplementary material for: The Microbial Diversity of Traditional Spontaneously Fermented Lambic Beer
Source: PLoS One. 2014 Apr 18;9(4):e95384. doi: 10.1371/journal.pone.0095384 (PMC3991685; doi:10.1371/journal.pone.0095384)
Supplement: Table S2 — Overview of MALDI-TOF MS clusters and the identifications of the representative isolates. The number of isolates in each MALDI-TOF MS cluster is given in parentheses. The accession number of the cluster representative sequence is given when sequence similarity with a known sequence was below 100%. B: bacterial MALDI-TOF MS cluster, Y: yeast MALDI-TOF MS cluster. (PDF) [file pone.0095384.s005.pdf]

MALDI-TOF MS cluster identifier	Strain number of representative isolate	Accession number*	Sequence type	Identification	Accession number highest hit	Similarity
B-1 (1)[a]	LMG 27882			*Gluconobacter cerevisiae*		
B-2 (43)	R-47374; R-49023	KJ186115	*rpoB*	*Enterobacter hormaechei*	AJ543724	99%
B-3 (159)	R-47368; R-47377; R-49018	KJ186116	*rpoB*	*Enterobacter kobei*	JX494753	99%
B-4 (21)	R-49013		*rpoB*	*Raoultella terrigena*	KF057939	100%
B-5 (32)	R-49012	KJ186117	*rpoB*	*Citrobacter gillenii*	KF057931	99%
B-6 (194)	R-49008	KJ186118	*rpoB*	*Klebsiella oxytoca*	AJ871804	97%
B-7 (102)	R-49019;	KJ186120	*dnaJ*	*Escherichia/Shighella*	AB272648	98%
	R-49020	KJ186119	*rpoB*	*Escherichia/Shighella*	EU010107	99%
B-8 (387)	R-47375; R-47380-R-47386; R-49024-R-49031; R-49033-R-49039; R-49555		16S rRNA gene	*Hafnia paralvei*	FM179943	100%
B-9 (51)[b]	LMG 27440			*Acetobacter lambici*		
B-10 (314)	R-49097; R-49102		*pheS*	*Pediococcus damnosus*	AM899820	100%
Y-1 (16)	R-49565; R-49568; R-49827		D1/D2 26S rRNA gene	*Priceomyces carsonii*	U45743	100%
Y-2 (14)	R-49569; R-49824; R-49826		D1/D2 26S rRNA gene	*Wickerhamomyces anomalus*	U74592	100%
Y-3 (255)	R-49830; R-49831		D1/D2 26S rRNA gene	*Dekkera bruxellensis*	JQ689028	100%
Y-4 (111)	R-49654; R-49655; R-49662; R-49821; R-52120; R-52121		D1/D2 26S rRNA gene	*Saccharomyces cerevisiae*	JQ689017	100%
Y-5 (89)	R-49564; R-49820		D1/D2 26S rRNA gene	*Naumovia castellii*	HE576754	100%
Y-6 (299)	R-49562; R-49653; R-49661	KJ186121	*ACT1*	*Saccharomyces pastorianus*	ALJS01000103	99%
					ABPO01000006	99%
Y-7 (29)	R-49837; R-49838		ITS	*Kazachstania servazzii*	AY046153	100%
Y-8 (7)	R-49647; R-49648		D1/D2 26S rRNA gene	*Candida friedrichii*	HQ283384	100%
Y-9 (62)	R-49652; R-49844;	KJ186123	*ACT1*	*Debaryomyces hansenii*	CR382136	99%
	R-49570; R-49825	KJ186122	*ACT1*	*Debaryomyces hansenii*	CR382136	98%
Y-10 (7)	R-49650		*ACT1*	*Meyerozyma guilliermondii*	AJ389063	100%
Y-11 (3)	R-49567	KJ186127	D1/D2 26S rRNA gene	*Priceomyces sp.*	AB568341	99%
		KJ186126	ITS	*Priceomyces carsonii*	AJ586521	99%
Y-12 (5)	R- 49657		D1/D2 26S rRNA gene	*Dekkera anomala*	EF550258	100%
Y-13 (7)	R- 49649		D1/D2 26S rRNA gene	*Pichia membranifaciens*	EU057561	100%
Y-14 (1)	R-49839	KJ186128	D1/D2 26S rRNA gene	*Candida nemodendra*	EU011629	98%
Y-15 (1)	R-49840		D1/D2 26S rRNA gene	*Candida patagonica*	DQ841165	100%
Y-16 (1)[c]			D1/D2 26S rRNA gene	*Yarrowia lipolytica*	JQ689067	100%
Y-17 (2)	R-49843		D1/D2 26S rRNA gene	*Debaryomyces marama*	JN940502	100%

[a]Isolates of this cluster were characterized in a polyphasic taxonomic study as the new species *Gluconobacter cerevisiae* (Spitaels et al., 2013b).

[b]Isolates of this cluster were characterized in a polyphasic taxonomic study as the new species *Acetobacter lambici* (Spitaels et al., 2013a).

[c]Cluster Y-16 consisted of one isolate from the Cantillon brewery and three isolates from a second brewery. One of the latter isolates was chosen as representative for sequence-based identification.

*Accession numbers are given for the unique isolate sequences within the same MALDI-TOF MS cluster.
